# Troop education and avian influenza surveillance in military barracks in Ghana, 2011

**DOI:** 10.1186/1471-2458-12-957

**Published:** 2012-11-08

**Authors:** John Kofi Odoom, Samuel Bel-Nono, David Rodgers, Prince G Agbenohevi, Courage K Dafeamekpor, Roland M L Sowa, Fenteng Danso, Reuben Tettey, Richard Suu-Ire, Joseph H K Bonney, Ivy A Asante, James Aboagye, Christopher Zaab-Yen Abana, Joseph Asamoah Frimpong, Karl C Kronmann, Buhari A Oyofo, William K Ampofo

**Affiliations:** 1 Department of Virology, Noguchi Memorial Institute for Medical Research, University of Ghana, P.O. Box LG 581, Legon, Accra, Ghana; 2 Ghana Armed Forces Medical Directorate, Ministry of Defence, Accra, Ghana; 3 Veterinary Services Directorate, Ministry of Food and Agriculture, Accra, Ghana; 4 Game and Wildlife, Ministry of Lands and Natural Resources, Accra, Ghana; 5 U.S. Naval Medical Research Unit No.3, Cairo, Egypt

**Keywords:** Surveillance, Pandemic avian influenza, Biosecurity, Education, Military, Ghana

## Abstract

**Background:**

Influenza A viruses that cause highly pathogenic avian influenza (HPAI) also infect humans. In many developing countries such as Ghana, poultry and humans live in close proximity in both the general and military populations, increasing risk for the spread of HPAI from birds to humans. Respiratory infections such as influenza are especially prone to rapid spread among military populations living in close quarters such as barracks making this a key population for targeted avian influenza surveillance and public health education.

**Method:**

Twelve military barracks situated in the coastal, tropical rain forest and northern savannah belts of the country were visited and the troops and their families educated on pandemic avian influenza. Attendants at each site was obtained from the attendance sheet provided for registration. The seminars focused on zoonotic diseases, influenza surveillance, pathogenesis of avian influenza, prevention of emerging infections and biosecurity. To help direct public health policies, a questionnaire was used to collect information on animal populations and handling practices from 102 households in the military barracks. Cloacal and tracheal samples were taken from 680 domestic and domesticated wild birds and analysed for influenza A using molecular methods for virus detection.

**Results:**

Of the 1028 participants that took part in the seminars, 668 (65%) showed good knowledge of pandemic avian influenza and the risks associated with its infection. Even though no evidence of the presence of avian influenza (AI) infection was found in the 680 domestic and wild birds sampled, biosecurity in the households surveyed was very poor.

**Conclusion:**

Active surveillance revealed that there was no AI circulation in the military barracks in April 2011. Though participants demonstrated good knowledge of pandemic avian influenza, biosecurity practices were minimal. Sustained educational programs are needed to further strengthen avian influenza surveillance and prevention in military barracks.

## Background

Since the first human outbreak of the highly pandemic avian influenza (HPAI) H5N1 subtype in Hong Kong in 1997 [1,2], surveillance efforts to detect the H5N1-subtype virus in birds have increased. A risk factor of contracting the virus is by handling infected poultry and poultry products [3]. Between December 2003 and April 2007, an unprecedented epizootic of HPAI virus affected poultry and wild birds in over 50 countries on 3 continents [4]. In Africa, the H5N1 virus first appeared in Nigeria, followed by Egypt in 2006, and then spread rapidly to poultry farms in other African countries [5,6]. There has been limited spread of H5N1 from human to human, but military populations are especially susceptible to rapid spread of respiratory pathogens such as influenza [7]. In many developing countries, military personnel live in barracks in close proximity to poultry populations. These militaries have risk factors for both the transmission of zoonotic diseases and conditions favorable for rapid human to human spread, making them important groups for avian influenza surveillance and prevention efforts. The possibility of emergence of pandemic avian influenza in the military population that may be called upon to play a significant role in the pandemic response, presents a major public health challenge and biosecurity threat. The establishment of H5N1 in domestic poultry, unregulated trade of potentially infected poultry, and high-risk farming practices continue to put some African countries at high risk [8]. This increasing risk to the African region has highlighted the importance of African pandemic preparedness plans and their potential shortcomings [9].

As a result, interviews on knowledge and attitudes of poultry workers and educational interventions for health workers have been conducted. Studies in Europe and Asia have shown that risk perception in relation to avian influenza was high [10,11]. In a similar study in Oyo state, Nigeria, farm workers were knowledgeable about AI and its associated risks, poor poultry handling practices were observed [12].

In 2005, a pandemic preparedness plan for Ghana was drafted and approved to clearly define the actions and resources necessary to build the capacity in the country to adequately prepare for and respond to the threat of pandemic avian influenza by strengthening existing structures and their capabilities [13]. A year later the first avian influenza outbreak in Ghana occurred on April 14, 2007 at Kakasunanka near Tema, where 12,811 birds died and 23,327 were culled. The second outbreak occurred in Sunyani on May 11, 2007 with 210 deaths and 2,671 birds culled. Then on June 13, 2007, another outbreak occurred in Aflao where 350 birds died and 1,357 were culled [14,15]. All the outbreaks were close to military barracks. No human case was recorded in any of the 3 outbreaks.

Following the outbreaks in Ghana, there have been two simulation exercises with all stakeholders including the Ghana Police Service, Customs Excise and Preventive Services, Ghana Prisons Services, National Disaster Management Organization (NADMO), and the Ministry of Health (MOH), but not the Ghana Armed Forces (GAF).

The recent pandemic of novel influenza A/H1N1 2009 virus clearly illustrates the unpredictable nature of pathogens that require dynamic and evolving public health strategies for surveillance, disease management and mitigation. Militaries are called upon for pandemic response and leaders require the knowledge, skills, and experience to address the evolving nature of threats to public health. Educating military professionals to understand, monitor, respond to, control and prevent emerging infections is essential for the GAF. The GAF has the mission of providing security and protection to the citizens, and stability during times of crisis, such as pandemics. The presence of individual and commercial poultry farms in military barracks and the deployment of Ghanaian troops for international peacekeeping operations put troops and families at increased risk. A recent bird census in all garrisons of the GAF found 7,424 birds being kept in or near the garrisons, with fowl constituting 5,990 (81%) of all birds; followed by ducks and turkeys at 8% and 6%, respectively. To date, there are limited data on the knowledge, attitudes and practices of troops handling or living near these birds which may influence the potential for avian influenza transmission to humans. A public health campaign on avian influenza was implemented which included education of GAF troops who may own or handle poultry. Veterinarians also carried out AI surveillance by sampling domesticated birds in the military barracks.

## Methods

### Operational area

**Figure 1:**
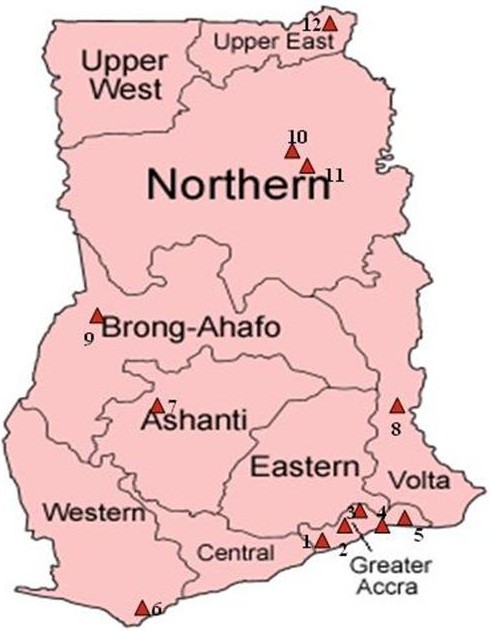
**Regional map of Ghana showing the distribution of military barracks visited. **The locations are 1; 5BN, 2; 49 Engineer Regiment, 3; 1BN, 4; Shai Hills, 5; Asutuare, 6; 2BN, 7; 4BN, 8; 66 Artillery, 9; 3MRS, 10; Airforce, 11; Airborne, 12; Bawku.

The exercise took place in 12 Ghana Armed Forces barracks in the country from 7 to 28 April 2011. These barracks are located in the coastal, tropical rain forest and northern savannah belts (Figure 1).

### Education

Seminars on pandemic avian influenza were conducted to sensitize all ranks of GAF personnel from the Army, Navy and Air Force, civilian employees of the Ministry of Defense, spouses and dependants of the uniformed personnel and civilians living within or near the catchment areas of the military barracks. The educational seminars were held in all garrisons. Attendance sheets were provided at the entrance of seminar rooms for the attendees to register their names, units, ranks and signatures. Within the Western region, the Army, Navy and Air Force were brought together at the second battalion (2BN) facilities for the seminar, while in the far north, troops from detachments in Bazua and Bawku came together for the seminar in Bawku. In the Northern region, separate seminars were held for the Air Force and the Airborne Force at the Barwah Barracks.

Education was done in four parts; showing a dramatization of a hypothetical pandemic, conducting didactic lectures, showing an AI biosecurity educational program, and question and answer sessions. The dramatization “Fatal Contact,” was developed in the US and depicts an avian influenza pandemic starting in China and spreading to the US and Africa, leading to many deaths. The didactic lectures were conducted by military and civilian veterinarians, public health officials and scientists on the different types of avian influenza viruses, factors contributing to emerging and re-emerging zoonotic diseases, signs and symptoms of bird influenza, surveillance techniques, outbreaks and outbreak zoning. An AI biosecurity video, developed by the Canadian Poultry Feed Agency, was screened. The final part was an interactive section where the troops were allowed to ask questions and were answered by the resource persons.

### Sampling

Poultry production in Ghana can be classified into three categories by installed capacity (bird population), marketing system and level of integration of its operations. These categories include commercial farms, semi-commercial farms and backyard/village poultry producers. On animal health services delivery, the GAF has one veterinary clinic that provides regulatory, field and clinical services in the capital city Accra.

A cross sectional study using an active avian influenza surveillance approach was conducted within 12 military barracks. A convenience sampling method based on the availability and consent of a household member was applied. Where a member or owner of the household was available and consented to the study, a maximum of 30-60 minutes was allotted for animal sampling and the individual interviewed. Households were then classified according to the installed capacity. Using criteria for eligibility based on birds whether apparently healthy or with respiratory signs or gastroenteritis or nervous illness, subjects were conveniently selected for either tracheal or cloacal swabbing. Verbal consent was obtained from all poultry owners to take swabs from their birds. Backyard poultry owners and household members were interviewed. A semi-structured questionnaire was administered and information on demographics, basic hygiene practices, quantity of poultry owned, poultry death reporting, practices when deaths occurred, knowledge of the cause of death, and knowledge of avian influenza was collected.

A total number of 680 birds from 102 households were sampled. At Teshie and Burma Camp both tracheal and cloacal samples were taken from each bird, while at the other sites cloacal samples were taken from ducks and tracheal samples from any other birds. All samples collected were appropriately labelled and stored in dry shippers containing liquid nitrogen and transported to the National Influenza Centre (NIC) at the Noguchi Memorial Institute for Medical Research (NMIMR) for processing. At the laboratory, all samples were transferred to -70°C for storage until ready for processing.

### Sample processing

Processing of samples took place in the Biosafety level-3 laboratory. Samples from 14 fowls found with pox-like lesions and respiratory abnormalities were separated and processed. The remaining samples were pooled according to swab site (tracheal or cloacal), bird type and household. The pools ranged from a single sample to 11. In all 94 pools obtained, there were 58 tracheal, 35 cloacal and 1 feather thallus (domesticated wild bird) samples.

### RNA extraction

Viral RNA was extracted from 140 μl of pooled or individual avian tracheal or cloacal swabs using the QIAmp viral RNA mini kit (Qiagen, Hilden, Germany) according to the manufacturer’s instructions. RNA was eluted in 60 μl of elution buffer and 8 μl used as template for real time Reverse Transcription-Polymerase Chain Reaction (rRT-PCR).

### Real time RT-PCR

Two rRT-PCR protocols described by the Centers for Disease Control and Prevention (CDC), Atlanta, Georgia, USA and Spackman et al., 2002 for influenza viruses, were used to screen all the samples [16,17]. RNA was amplified by rRT-PCR using the AgPath-ID One-Step RT-PCR Kit (Ambion, Austin, Texas, USA) in a 25 μl reaction mixture or Qiagen One Step RT-PCR Kit (Hilden, Germany). The RT step conditions for all primer sets were 30 min at 50°C and 15 min at 94°C. A two-step PCR cycling protocol was used for the matrix gene primer set as follows: 45 cycles of 94°C for 0 sec and 60°C for 30 sec and for the AgPath-ID as 45 cycles of 95°C for 2 min and 55°C for 30 sec. All temperature transition rates were set at the maximum transition rate of 20. Fluorescence data were acquired at the end of each annealing step.

The rRT-PCR assays were performed on real time PCR instruments (Applied Biosystems, Singapore) with SDS software version 1.4 (Applied Biosystems, Singapore). Results of the rRT-PCR assays were determined by the analyses of cycle threshold values generated by SDS auto analysis on samples against reference positive and negative controls.

### Data analysis

Demographic data was entered in an electronic database file (Microsoft Excel, 2003). Basic analyses were performed using Microsoft Excel to generate frequencies, graphs and tables.

### Ethical approval

Military to Military influenza surveillance is considered a public health activity and is conducted with the Ghana Armed Forces Health Directorate, Veterinary Services Directorate and Ghana Health Service in a manner consistent with Ghanaian standards. It has served to facilitate identification of potential sources of avian influenza around military bases, through educational seminars, and active and passive processing of bird samples. This activity is under the Surveillance for influenza virus in acute respiratory illness in Ghana project which was initiated in 2007 under the integrated disease surveillance and response system of the Ghana Health Service with ethical clearance 015/06-07 from the Noguchi Memorial Institute of Medical Research Institutional Review Board.

## Results

Of the 1028 persons that attended the educational seminars, 674 (56.4%) were males and 354 (43.6%) females. The highest attendance of 200 (19.5%) was recorded in 3 Infantry Battalion, Sunyani, followed by Burma Camp with 150 (14.6%) while Bawku recorded the least at 31 (3.0%) as shown in Table 1. The highest male (84.8%) and female (52.3%) attendance were recorded in 66 artillery in the Volta region and Michel Camp respectively. In all the barracks with the exception of the barracks in the Northern and Upper East regions, spouses and dependents of troops attended the troop education program. From the survey, 668 (65%) participants had heard about avian influenza but did not know the difference between avian influenza H5N1 and pandemic A H1N1 2009 influenza virus. One hundred and fifty-four (15%) participants reported preparing sick and dead chicken for food and 627 (61%) reported lack of knowledge in signs and symptoms of avian influenza. Daily cleaning of pens was commonly reported among the troops, however, basic hygiene practices like hand washing before and after attending to poultry was rarely observed. Birds with fowl pox lesions and respiratory abnormalities were mixed with healthy birds.

**Table 1:** Attendance of participants during troop education in military barracks

**Region**	**Garrison**	**Barracks**	**Attendance**				
			**Male**	**%**	**Female**	**%**	**Total**
Greater Accra	1 & 5	Teshie	75	80.6	18	19.4	93
		Burma Camp	85	56.7	65	43.3	150
		Michel Camp	57	47.5	63	52.5	120
Volta	7	66 artillery	89	84.8	16	15.2	105
Western	2	Myohaung Barracks (2BN)	75	60.5	49	39.5	124
Ashanti	4	Uadara (4BN)	62	62	38	38	100
Brong Ahafo	3	Liberation Barracks (3BN)	116	58	84	42	200
Northern	6	Airforce	34	72.3	10	27.7	44
		Airborne	50	82	11	18	61
Upper East		Bawku/Bazua	31	100	0	0	31
Total			674		354		1028

**Figure 2:**
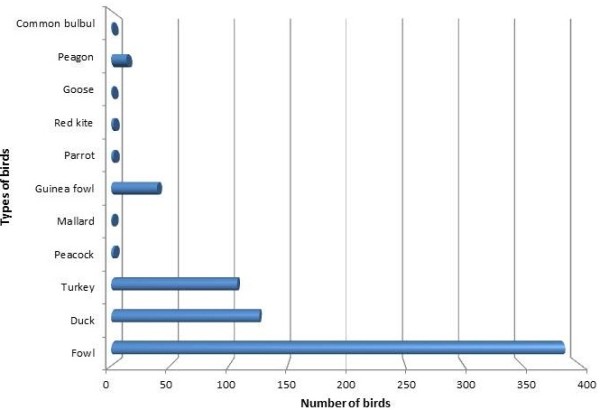
Various species of birds found in backyard poultry in military barracks, Ghana.

**Figure 3:**
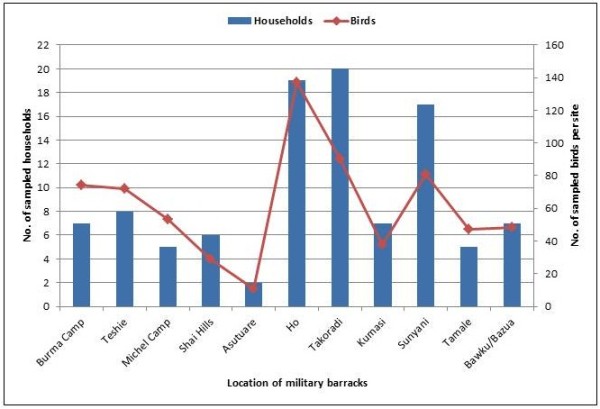
Number of birds sampled from households within the Military barracks.

A total of 828 samples made up of 579 tracheal, 248 cloacal and one feather thallus were sampled from 102 households (Table 2). The different bird species sampled are shown in Figure 2. Every site and household had more fowls (*Gallus gallus domesticus*) than the other bird species 386 (56.7%). One hundred and twenty-five (18.4%) ducks (*Anas platyrhyncos domesticus*) and 108 (15.9%) turkeys (*Meleagris gallopavo*) were sampled. However, no ducks or turkeys were found in garrisons three and five respectively. Some wild birds like the common bulbul (*Pycnonotus barbatus*), parrot (*Psittacus erithacus*), mallard (*Anas platyrhyncos domesticus)* and black kite (*Milvus migrans*) were kept by individuals as pets in 2BN in Takoradi. Of the 102 households sampled, the highest numbers, 19 (19%) each, were from 7 garrison in Ho and 2BN in Takoradi while the lowest number of 2 (2%) was from Asutsuare (Figure 3). Almost all poultry were free ranging and mixing of poultry from different households with wild birds was common. Fowl pox lesions and respiratory abnormalities were observed in 4% of the fowl.

**Table 2:** Regional bird population census and bird sampled in the military garrisons

**Region**	**Garrisons**	**Barracks**	**Bird population**	**House-holds**	**No. Birds sampled**	**Sampling rate**
Greater Accra	5	Burma Camp	1011	7	74	7%
		Teshie	125	8	72	58%
	1	Michel Camp	1174	5	53	5%
		Shai Hills	120	6	29	24%
		Asutuare	22	2	11	50%
Western	2	Myohaung	404	19	90	22%
Brong Ahafo	3	Sunyani	819	17	81	10%
Ashanti	4	Kumasi	68	7	38	56%
Northern	6	Tamale	1071	5	47	4%
Volta	7	66 artillery	498	19	137	28%
Upper East	+Detachment	Bawku/Bazua	498	7	48	10%
Total			5810	102	680	12%

+Troops from the Airborne in the 6BN, Tamale, stationed in Bawku and Bazua.

None of the samples from the sick or healthy fowls were positive for influenza A using the two real-time RT-PCR protocols.

## Discussion

Surveillance of avian influenza by Veterinary Services Directorate (VSD) started in Ghana in 2006 with the focus on commercial farms, live bird markets and backyard farms [18]. This was preceded by the pandemic preparedness plan by the National Avian Influenza Working Group (AIWG) in 2005 to detect and respond to threats of pandemic avian influenza. To date few studies aiming at identifying the HPAI H5N1 in poultry have been conducted after the 2007 pandemic avian influenza outbreak in the country. We present a report on education and avian influenza surveillance conducted in military barracks across the country. The Military personnel and their dependents fully participated in the seminars. Participants were taken through the transmission, clinical signs and symptoms, epidemiology, pathogenesis and public health importance of AI and biosecurity. It was observed during the seminars that dependent female spouses asked most of the questions, which could be attributed to reservations of uniformed personnel to ask questions in front of superiors, or to the greater role of dependent spouses in care of backyard poultry. One of the most frequently asked questions in the barracks was about the difference between human and avian influenza viruses. Participants also wanted to know whether vaccination against H1N1 protects against H5N1 and whether touching or eating well-cooked infected birds was safe. These questions necessitate the need for continuing education on avian influenza and ways to recognize disease and prevent spread for frequently rotating troops and their dependents. Our findings showed that participants in all the barracks have good awareness of pandemic influenza, but detailed knowledge of some important facts was less well known. The heightened awareness is expected due to the recent 2009 pandemic H1N1 global outbreak that led to mass education programs. This finding was similar to what was seen in Italy [19]. However, in spite of the high awareness and widespread knowledge of AI and personal protection measures in the public, most officers of the GAF and their families still practice unsafe poultry handling. Direct contact with poultry and poultry products was common among household members of the military. Reporting of bird death to veterinary officers was low and in some circumstances people did not know where to report cases. Fifteen percent of participants admitted they still prepare sick and dead poultry for household consumption. These findings provide evidence that awareness does not necessarily lead to behaviour change. Behaviour change involves comprehensive and multidisciplinary intervention, which combines communication, feasible and practical recommendations, including economic considerations. In this context, improvement in risky practices can only be achieved through relentless behaviour change efforts. While knowledge that disease can spread from sick birds to humans is common, education is needed on how to minimize risk of disease spread amongst bird populations, and from birds to humans, as well as understanding what to do with sick birds. Our observations are similar to those by Vathsala and Petticrew [20,21] that continuous education and training is a process of updating knowledge, developing skills, bringing about attitudinal changes, and improving the knowledge and skills of troops who may be called upon in a pandemic to perform their tasks efficiently and effectively.

Some limitations of the study exist. The difference in knowledge before and after a lecture was not examined, nor was the decay of knowledge 3 months or 6 months after participating in the training assessed. Selection of households was also based on the availability and consent of the owners. Where households owners were not present and consent not given, the house was left out. For each selected household, not more than 5 birds per species were sampled from the same hen coop or farm.

During sampling, 680 birds from 11 different species were sampled and tested for influenza A virus. Of these, fowls were the majority and most common in every household. All birds were physically well except for 14 fouls that were found sick with fowl pox and respiratory abnormalities. None of the birds were found positive for influenza A using two rRT-PCR protocols [16,17]. The results obtained from the survey showed no evidence of the presence of avian influenza virus (AIV) in the birds sampled within the military barracks across the country. The results are consistent with similar studies carried out in commercial farms, backyard farms, live bird markets and wild birds in the Tema metropolis from May 2009 to September 2010 [14,15,22,23]. In spite of high risk practices within the military camps, no positive birds have been found and there have been no reports of outbreaks. However, practices found among poultry farmers in the barracks suggest outbreaks may rapidly spread if disease is introduced at some point. Basic hygiene like washing of hands before and after handling poultry was found lacking in some barracks. In addition, personal protection such as rubber gloves and nose masks used to work safely with poultry on a daily basis was virtually absent. Furthermore, farm management practices, particularly fencing of farms and establishment of foot baths should be implemented. The mingling of wild birds with backyard poultry poses imminent danger to naive poultry through interspecies transmission of influenza and other pathogens.

The GAF is an important part of the biosecurity and pandemic response for Ghana. Like many militaries, the GAF is also susceptible to outbreaks of respiratory disease such as influenza, endangering their ability to provide stability in times of crisis. The actions and findings described in this paper are important in several respects. Firstly, education and awareness of avian influenza among troops and their dependents were improved. Secondly, public health leaders obtained a better understanding of the challenges and barriers by visiting all garrisons and conducting seminars and sampling. Even though no avian influenza was found to be circulating at the time of the activities, poultry farming practices favorable to the spread of disease once introduced were identified. Future efforts should target better education and equiping poultry farmers to prevent the spread of disease among birds and between birds and humans. Some interventions, like improved hand washing after handling poultry and better reporting of sick birds to appropriate authorities, can be improved primarily by education. Others, such as improved facility designs and provision of protective equipment will require more investment. Coordination between the GAF and the NIC was strengthened by these exercises, and young military veterinarians and public health workers gained experience. The authors hope that the insights gained into the vulnerabilities and challenges facing the GAF, an integral part of the Ghana healthcare and pandemic response system, will better enhance future public health efforts of the GAF and the larger national and global public health communities.

## Conclusion

Our results provide new insights on poultry handling practices with regard to avian influenza by the GAF. Our surveillance found no evidence of AI circulation in poultry in military barracks during the study period. Despite a high level of awareness, the troops and their families were unaware of appropriate biosecurity measures to reduce risk of spread of disease within avian populations or between birds and humans. Practices to minimize the transmission and spread of disease are not routinely followed and need to be strengthened. This should include avoidance of direct contact with sick or dead poultry, and use of protective equipment such as gloves and masks when contact is unavoidable.

## Competing interests

The authors declare that they have no competing interests.

## Authors’ contributions

JKO participated in the troop education, surveillance and the manuscript writing, SBN was involved in study design and editing of the manuscript, CKD was involved in study design and interpretation of results, RMLS participated in analysis of results and editing of the manuscript, RS participated in troop education and surveillance, RT participated in the troop education, surveillance and the manuscript writing, FD participated in the surveillance, analysis of samples and manuscript writing, JHB participated in sample analysis and writing of the manuscript, IAA participated in sample analysis and writing of the manuscript, JA participated in AI surveillance and sample analysis, CZYA participated in AI surveillance and sample analysis, PGA participated in troop education, surveillance and the manuscript writing, DR participated in troop education, surveillance and manuscript writing. KCK contributed to data interpretation and the manuscript writing, BAO contributed to data interpretation and the manuscript writing and WKA supervised the design and implementation of the study protocol. All authors read and approved the final manuscript.

## Authors’ disclaimer statement

The views expressed in this article are those of the authors and do not necessarily reflect the official policy or position of the Department of the Navy, Department of Defense, nor the U.S. Government, nor the Ghana Armed Forces nor the Government of Ghana.

## Copyright assignment statement

Samuel Bel-Nono, David Rodgers^3^, Prince G. Agbenohevi^3^, Courage K. Dafeamekpor, Roland M. L Sowa are Ghana Armed Forces personnel. This report was prepared within the conduct of their official work.

LCDR Karl C. Kronmann and CAPT Buhari Oyofo are military service members. This work was prepared as part of their official duties. Title 17 U.S.C. §105 provides that ‘Copyright protection under this title is not available for any work of the United States Government’. Title 17 U.S.C. §101 defines a U.S. Government work as a work prepared by a military service member or employee of the U.S. Government as part of that person’s official duties.

The authors do not have a commercial or other association that might pose a conflict of interest. This research was supported by the Ghana Armed Forces Medical Directorate, World Health Organization, Centers for Disease Control and Prevention, Atlanta, Georgia, USA, US Naval Medical Research Unit No.3, Cairo, Egypt, the Global Emerging Infections Surveillance and Response System of the U.S. Armed Forces Health Surveillance Center, Noguchi Memorial Institute for Medical Research and Ghana Health Services.

The findings and conclusions in this report are those of the authors and have not been presented elsewhere.

## Pre-publication history

The pre-publication history for this paper can be accessed here:

http://www.biomedcentral.com/1471-2458/12/957/prepub
